# Targeted stool metabolomics suggests exploratory catecholamine- and tryptophan-linked metabolic features in autism spectrum disorder

**DOI:** 10.3389/fnins.2026.1858005

**Published:** 2026-07-03

**Authors:** Kevin Liu, Huixi Li, Shaohan Zhang, Muya Xi, Junru Zhu, Jonathan Chen, William Xu, Alexander Xie, Alexandros Makriyannis, Jason J. Guo, Xue-Jun Kong

**Affiliations:** 1 Fetal-Neonatal Neuroimaging and Developmental Science Center, Division of Newborn Medicine, Boston Children’s Hospital, Boston, MA, United States; 2 Department of Pharmaceutical Sciences, Center for Drug Discovery, Northeastern University, Boston, MA, United States; 3 Athinoula A. Martinos Center for Biomedical Imaging, Massachusetts General Hospital, Charlestown, MA, United States; 4 Department of Chemistry and Chemical Biology, Barnett Institute for Chemical and Biological Analysis, Northeastern University, Boston, MA, United States; 5 Department of Medicine and Psychiatry, Beth Israel Deaconess Medical Center, Boston, MA, United States

**Keywords:** autism spectrum disorder, biomarkers, catecholamines, gut-brain axis, machine learning, microbiome, stool metabolomics, tryptophan metabolism

## Abstract

**Background:**

Gut-brain axis dysregulation and microbiome-linked metabolic alterations have been implicated in autism spectrum disorder (ASD), but the contribution of gut-derived neuroactive metabolites remains incompletely characterized.

**Methods:**

We conducted a cross-sectional case-control study of 59 participants (32 ASD, 27 controls) and quantified 18 stool metabolites related to catecholamine synthesis, inhibitory neurotransmission, and tryptophan-linked NAD+-precursor metabolism using targeted liquid chromatography-tandem mass spectrometry. Group differences were assessed using fold-change analysis and linear models adjusted for age and sex. Random forest models evaluated classification performance, and within-group Spearman correlations were used to examine metabolic relationships.

**Results:**

Norepinephrine showed the largest increase in ASD, whereas dopamine and tetrahydrobiopterin exhibited nominal group differences that did not remain significant after correction for multiple testing. A three-metabolite panel comprising tetrahydrobiopterin, γ-aminobutyric acid, and kynurenine showed exploratory discrimination between groups (area under the receiver operating characteristic curve = 0.750, 95% confidence interval 0.622–0.878), but this performance requires external validation. Correlation analysis revealed conserved bile acid coupling in both groups. In controls, tryptophan was positively associated with kynurenine, whereas this relationship was not observed in ASD. Instead, ASD samples showed broader associations between tryptophan and metabolites linked to neurotransmission and NAD+-precursor metabolism.

**Conclusion:**

Stool metabolite profiling revealed altered organization of tryptophan- and catecholamine-linked metabolic associations in ASD and identified a small metabolite panel with exploratory discriminative potential. These findings provide a foundation for future studies examining gut-derived neuroactive metabolites in ASD and their relationship to gut-brain axis biology.

## 1 Introduction

Autism spectrum disorder (ASD) is a heterogeneous neurodevelopmental condition characterized by impairments in social communication and restricted, repetitive behaviors (American-Psychiatric-Association, 2013). Considerable variability across behavioral, cognitive, and biological domains contributes to the diagnostic complexity of ASD (Feczko et al., 2018; Luo et al., 2020; Mosconi et al., 2023). Because diagnosis relies entirely on behavioral assessment (Lord et al., 1994, 2000; Parellada et al., 2023), identifying objective, biologically grounded biomarkers remains a major priority for improving early detection and clinically meaningful subtype stratification. ASD has a substantial genetic contribution, with risk influenced by rare variants, common polygenic variation, copy-number variants, and gene-environment interactions (Rylaarsdam and Guemez-Gamboa, 2019; Havdahl et al., 2021). However, genetic findings do not fully explain the clinical and biological heterogeneity of ASD, motivating complementary studies of downstream molecular phenotypes, including metabolism, immune signaling, and host-microbiome interactions (Havdahl et al., 2021).

The gut-brain axis (GBA) has emerged as a promising area for ASD biomarker discovery, as it encompasses bidirectional communication between the gut and central nervous system and integrates neural, immune, endocrine, and metabolic signaling pathways that have been proposed to contribute to ASD-related features (Carabotti et al., 2014; Petrut et al., 2025). Accumulating evidence suggests that dysbiotic microbiota are associated with ASD and may contribute to aspects of its underlying biology, potentially through corresponding changes in metabolites. For instance, the restoration of gut microbiota in an idiopathic mouse model of ASD exhibiting autism-like behaviors through human fecal microbiota transplantation has been shown to alleviate social deficit symptoms of ASD, while also normalizing the colon and plasma metabolomes (Zheng et al., 2024). Parallel evidence involving microbiota transfer therapy in children with ASD has shown persisting improvements in both gastrointestinal (GI) and behavioral symptoms of ASD and a convergence of plasma and fecal metabolome profiles toward those of typically developing children (Kang et al., 2017, 2020). These convergent findings suggest that gut microbial composition and its metabolic activities are associated with gastrointestinal and behavioral outcomes in ASD, consistent with a potential role in gut-brain axis signaling. Several gut-derived metabolites have been implicated in ASD and provide mechanistic motivation for targeted assays. For example, treatment with Limosilactobacillus reuteri increases gut tetrahydrobiopterin (BH4) and restores social behavior in genetic ASD mouse models (Buffington et al., 2021; Dooling et al., 2022). Furthermore, BH4 is also an essential cofactor for tryptophan hydroxylase, the rate-limiting enzyme in serotonin synthesis, thereby linking pterin metabolism to the serotonin/N-acetylserotonin/melatonin pathway; this pathway is particularly relevant to ASD because whole-blood hyperserotonemia remains among the most consistently replicated peripheral biomarkers reported in ASD, and altered melatonin-related phenotypes have also been described (Pagan et al., 2014; Muller et al., 2016; Fanet et al., 2021; Eichwald et al., 2023; Esposito et al., 2024). Catecholamine-related biology has been implicated in ASD across central and peripheral studies, while gut microbes may also influence catecholamine availability and signaling (Launay et al., 1987; Pavãl, 2017; Miri et al., 2023). However, evidence varies by biospecimen and does not yet establish a consistent stool catecholamine signature in ASD (Launay et al., 1987; Needham et al., 2021) γ-Aminobutyric acid (GABA) has been linked to ASD behavior and microbial composition (Wang et al., 2025), and altered tryptophan (Trp) metabolism has been reported in ASD across several biospecimens, including studies of kynurenine (KYN)-pathway metabolites, although findings are heterogeneous across cohorts and matrices (Almulla et al., 2023; Yildirim et al., 2023; Santana-Coelho, 2024). Together, these findings highlight a gut-derived metabolic network linking microbial activity to host neurochemical balance (Vuong and Hsiao, 2017; Hu et al., 2020).

The targeted metabolite panel was selected *a priori* to cover three biologically motivated domains relevant to gut-brain axis signaling in ASD: catecholamine synthesis and regulation, inhibitory neurotransmission and neuromodulatory signaling, and tryptophan-related metabolism including kynurenine- and NAD+-precursor metabolites (Vuong and Hsiao, 2017; Needham et al., 2021; Peralta-Marzal et al., 2021). Specifically, BH4, dopamine (DA), and norepinephrine (NE) were included to evaluate catecholamine-linked metabolism, based on prior evidence implicating BH4-dependent monoamine biology, dopaminergic signaling, and microbial catecholamine activity in ASD-relevant phenotypes (Pavãl, 2017; Buffington et al., 2021; Miri et al., 2023). GABA and endocannabinoid-related metabolites were included to capture inhibitory and neuromodulatory signaling relevant to ASD and gut-brain communication (Zamberletti et al., 2017; Aran et al., 2019; Braga et al., 2024; Wang et al., 2025). Trp, KYN, nicotinic acid (NA), and related metabolites were included to assess Trp-linked and NAD+-precursor relationships, given prior evidence implicating tryptophan-kynurenine metabolism in ASD and microbial participation in NAD+ precursor cycling (Chellappa et al., 2022; Almulla et al., 2023; Yildirim et al., 2023; Santana-Coelho, 2024). The panel was therefore intended to test a focused mechanistic hypothesis rather than to provide untargeted metabolome coverage. In this study, we hypothesized that metabolites linked to gut microbiome dysbiosis would exhibit coordinated alterations in ASD and serve as characteristic features of the ASD fecal metabolome. We therefore applied a hypothesis-driven, targeted liquid chromatography-tandem mass spectrometry (LC-MS/MS) approach to quantify gut-derived metabolites previously implicated in GBA signaling. The resulting profiles offer a biochemical window into gut-linked neurochemical processes and may inform future evaluation of mechanistically grounded candidate biomarker features.

## 2 Materials and methods

### 2.1 Participants and ethical approval

This was an observational, cross-sectional case-control study. The study was conducted in accordance with the ethical principles of the Declaration of Helsinki and approved by the Institutional Review Board of Massachusetts General Hospital (protocol 2022P001749) under a formal collaboration agreement with Northeastern University (agreement 2023A064744). Parents or legal guardians of all participants provided written informed consent, and child assent was obtained when developmentally appropriate.

Participants with ASD were recruited from Massachusetts General Hospital (Charlestown, Massachusetts, USA) based on the following inclusion criteria: (1) male or female participants aged 2.5–22 years; (2) confirmed clinical diagnosis of ASD; (3) no recent use of antibiotics, probiotics, or oxytocin within the previous 4 weeks; (4) stable medication regimen for at least 4 weeks prior to enrollment (medication use was screened to ensure stability but was not modeled as a covariate in downstream analyses); and (5) willingness to provide stool samples and participate in interviews and study procedures. The exclusion criteria included the following: (1) pregnancy (before or during the study); (2) comorbid neurological or psychiatric disorders such as bipolar disorder or history of substance use disorder, active cardiovascular disease not controlled by medication, or current use of psychotropic medications; and (3) treatment with oxytocin or probiotics within 4 weeks prior to sample collection.

Non-ASD controls (NAC) were recruited from the community and research registries and the inclusion criteria were: (1) male or female participants aged 2.5–22 years; (2) no diagnosis of ASD or other developmental disorder; (3) no recent use of antibiotics, probiotics, or oxytocin within the previous 4 weeks; (4) stable medication regimen for at least 4 weeks; and (5) willingness to provide stool samples and participate in study procedures. Exclusion criteria mirrored those for the ASD group, including pregnancy, comorbid neurological or psychiatric disorders, active uncontrolled cardiovascular disease, and recent treatment with oxytocin or probiotics.

ASD status was defined based on a confirmed clinical diagnosis documented in the medical record. Non-ASD controls were defined as individuals without a diagnosis of ASD or other developmental disorder. Age and sex were obtained from parent report and medical records. No interventions were administered as part of this study.

### 2.2 Chemicals and reagents

HPLC-grade methanol, acetonitrile, water, and formic acid (98%) were purchased from Fisher Scientific (Hampton, NH, USA). Internal standards, including deoxycholic acid-d4, tryptophan-d5, serotonin-d4, and dopamine-d2, were obtained from C/D/N Isotopes (Vaughan, ON, Canada). All 18 chemical metabolites of interest were acquired from Thermo Fisher or MilliporeSigma, except anandamide and 2-arachidonoylglycerol, which were prepared internally at the Center for Drug Discovery, Northeastern University. These compounds were first used to generate standard calibration curves on the mass spectrometer and were then prepared as quality-control (QC) mixtures to monitor instrument stability and reproducibility while analyzing the study samples.

### 2.3 Sample collection and preparation

Fresh fecal samples were collected from participants in fecal collection and storage tubes containing ethanol (95%) (OMNImet⋅GUT, DNA Genotek, Canada) and stored at −80 °C until analysis. For metabolomic profiling, samples were thawed at 4 °C and prepared in triplicate. Approximately 30 mg of fecal material was mixed with 400 μL of ice-cold methanol, homogenized at 25 Hz for 5 min, and centrifuged at 10,000 × *g* for 10 min at 4 °C. The supernatant was transferred to a clean tube and dried using a Labconco CentriVap concentrator (Labconco, Kansas City, MO, USA) for 40 min at 35 °C. The dried residue was re-suspended in 150 μL methanol:water (1:1, v/v), spiked with 10 μL of the internal standard mixture, and centrifuged again for 3 min at 10,000 × *g* (4 °C). Processed samples were stored at −80 °C until LC-MS/MS analysis.

### 2.4 LC-MS/MS analysis

LC-MS/MS analyses were performed at Northeastern University (Boston, MA, USA). Chromatographic separation was performed on a Vanquish UHPLC system coupled to a TSQ Altis triple-quadrupole mass spectrometer (Thermo Fisher Scientific, San Jose, CA, USA). The injection volume for all samples was 20 μL.

#### 2.4.1 Chromatographic conditions

Metabolites were separated using a Kinetex^®^ F5 column (3.0 × 150 mm, 2.6 μm) with a SecurityGuard™ ULTRA UHPLC F5 guard column, maintained at 30 °C. The mobile phase consisted of 0.1% formic acid in water (A) and 0.1% formic acid in acetonitrile (B). The gradient elution program was run at 0.7 mL/min as follows: 0–2 min, 10% B; 2–10 min, 5%–95% B; 10–12 min, 95% B; 12–13.5 min, 95%–10% B; and 13.5–15 min, 10% B for re-equilibration.

#### 2.4.2 Mass spectrometry conditions

The mass spectrometer was operated in both positive and negative electrospray ionization (ESI) modes using Thermo Fisher’s Advanced Active Ion Management (AIM) polarity-switching technology. Source parameters were optimized as follows: positive ion voltage 4,500 V; negative ion voltage 3,000 V; sheath gas 60 (arb); auxiliary gas 7 (arb); sweep gas 2 (arb); ion-transfer tube temperature 380 °C; vaporizer temperature 350 °C. Collision energies and fragment ions were optimized for each metabolite using authentic standards or predicted from molecular structures (Supplementary Table 1).

#### 2.4.3 Quality control

A quality-control (QC) dilution series was prepared from a mixture of all metabolites of interest at four concentrations to span the expected dynamic range: blank (solvent only), low QC (0.025 μM), medium QC (0.6 μM), and high QC (7.5 μM). QC samples were injected after every 40 study samples to monitor system stability, reproducibility, and analytical performance. Blank samples were used to assess background contamination and matrix effects.

### 2.5 Data processing

All quantified metabolite concentration data were processed using *MetaboAnalyst* (version 6.0) (Pang et al., 2024). For each biological sample, technical replicate intensities (*n* = 3) were first smoothed using kernel density estimation with a Gaussian kernel (bandwidth = 0.5) to reduce technical variability. These smoothed values were then averaged using the arithmetic mean. To ensure data quality, a filtering step was applied such that, within each sample, any metabolite feature with more than 33% missing values and a coefficient of variation (CV) greater than 1 across replicates was assigned a value of zero. After averaging, normalized data were obtained using a sequential pipeline comprising median normalization to account for inter-sample variability, log10 transformation to reduce right skew and compress the dynamic range, and Pareto scaling to emphasize moderate-intensity changes while retaining biological interpretability.

### 2.6 Statistical analysis

All statistical analyses were performed in *R* (version 4.4.2). Machine-learning workflows were implemented using the *tidymodels* framework (version 1.3.0), with random forest models fitted using the *ranger* engine (version 0.17.0). Classification performance metrics were computed using *yardstick* (version 1.3.2), and receiver operating characteristic analyses were conducted using *pROC* (version 1.19.0.1). Statistical testing was performed using *rstatix* (version 0.7.2). Pairwise correlations were computed using the *Hmisc* package (version 5.2.3). Data visualization was conducted using *tidyverse* packages. Unless otherwise specified, default parameters were used. All statistical analyses and data visualization are reproducible using the source code available at https://github.com/kevinliu-bmb/targeted-asd-stool-metabolomics.

Age was analyzed as a continuous variable, sex was analyzed as a binary categorical variable, and diagnostic group was coded as ASD versus non-ASD control. Factors were coded such that NAC and Female served as reference levels. For descriptive summaries, measurements below the limit of detection (LOD) were excluded. For inferential and machine-learning analyses, below-LOD values were retained as zeros in the normalized data matrix to preserve group structure. This approach was chosen because below-LOD observations were limited to a small subset of metabolites and manual inspection supported true low-abundance signal rather than sporadic technical failure. We therefore prioritized preserving the observed low-abundance structure rather than applying model-based imputation in a small dataset. However, we acknowledge that zero substitution can affect distributional properties and may attenuate or bias group differences.

#### 2.6.1 Differential abundance

Metabolite concentrations were median-normalized, log10-transformed, and Pareto-scaled as described above. Scaling was applied primarily for multivariate modeling to prevent high-abundance metabolites from dominating model training. Group differences were assessed using Wilcoxon rank-sum tests and linear models adjusted for age and sex. Log2 fold changes were derived from differences in log10-transformed means and converted to log2 scale by multiplying by log2(10). *P*-values were corrected using the Benjamini–Hochberg false discovery rate (FDR) method with significance defined as q < 0.05.

#### 2.6.2 Correlation analysis

Within-group associations were assessed using Spearman rank correlation coefficients. FDR correction was applied separately within each group across all tested metabolite pairs. Significant group-specific correlations were visualized using scatterplots with Spearman correlation coefficients and FDR-adjusted q-values.

#### 2.6.3 Machine learning

Random forest classifiers were constructed using 500 trees with default *mtry* and minimum node size parameters. No hyperparameter tuning was performed due to limited sample size. The machine-learning workflow was intended to estimate exploratory discriminative signal rather than to develop an optimized clinical classifier. Five-fold cross-validation stratified by diagnostic group was used to evaluate model performance. A fixed random seed (123) ensured reproducibility.

Model performance was assessed using area under the receiver operating characteristic curve (AUC), accuracy, sensitivity, specificity, precision, F1-score, and Brier score. Receiver operating characteristic (ROC) curves were generated using cross-validated predicted probabilities pooled across folds. AUC confidence intervals were estimated using 2,000 bootstrap resamples. ASD was defined as the positive class for all classification metrics. Class weights were not applied. Calibration was assessed using LOESS-smoothed curves comparing predicted probabilities to observed outcomes.

## 3 Results

**FIGURE 1:**
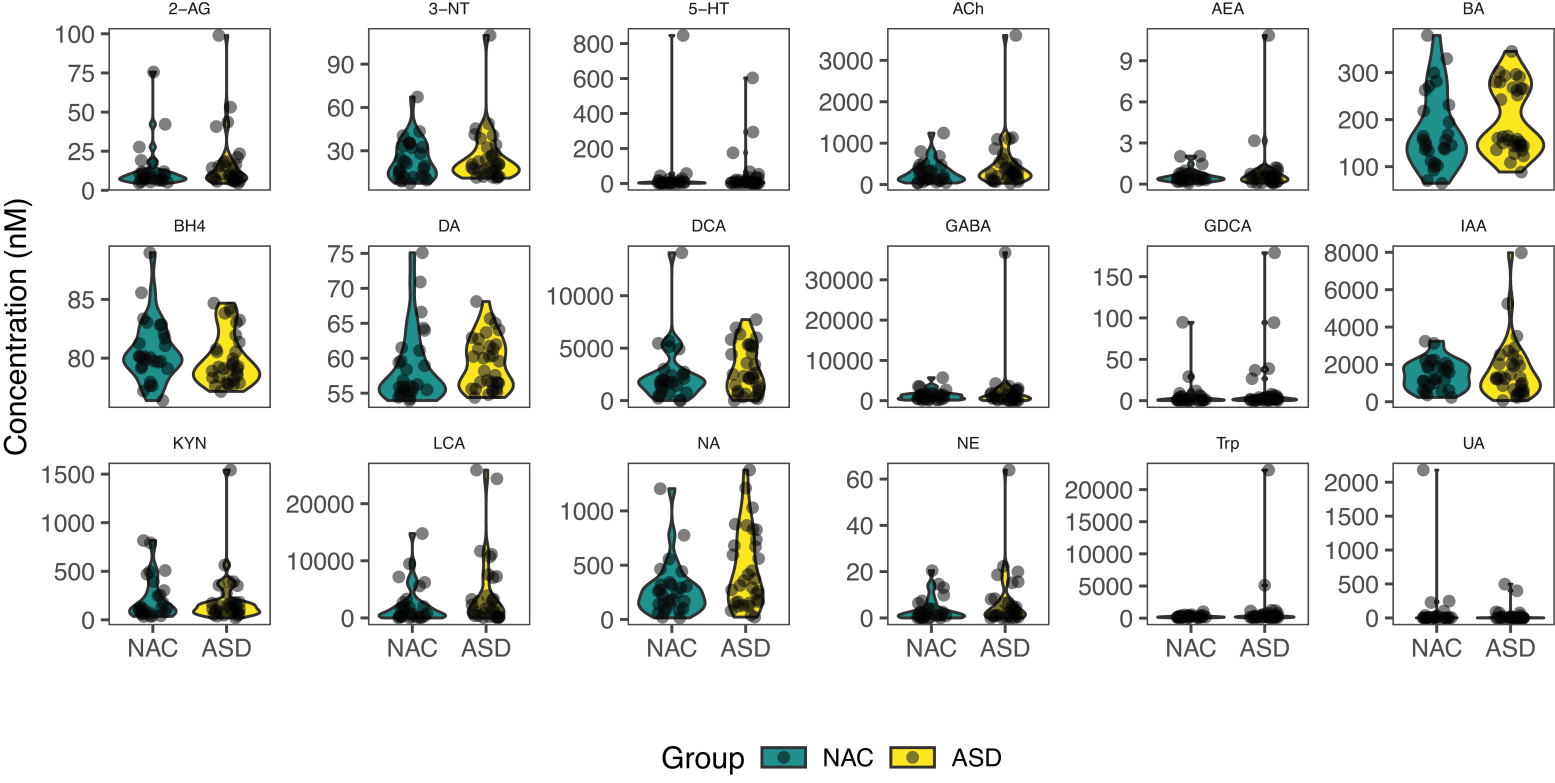
Distribution of metabolite concentrations in ASD and NAC groups.

The study included 59 participants: 32 individuals with ASD and 27 non-ASD controls. Participant characteristics are summarized in Table 1. The groups did not differ significantly in age (ASD: 6.86 ± 4.66 years; NAC: 8.57 ± 6.11 years; *p* = 0.322), but the sex distribution differed significantly, with a higher proportion of males in the ASD group (75.0%) than in the NAC group (37.0%; Fisher’s exact test, *p* = 0.00421; odds ratio = 4.94, 95% CI 1.47–18.21). The age distribution of participants by group is shown in Supplementary Figure 1. Measured fecal metabolites are summarized in Table 2, and their groupwise distributions are shown in Figure 1. For lithocholic acid (LCA), NE, and urolithin A (UA), some participant samples had replicate measurements below the limit of detection (LOD); these frequencies are also summarized in Table 2.

**TABLE 1:** Participant characteristics by diagnostic group.

	ASD (*n* = 32)	NAC (*n* = 27)	*p*-value
Age (mean ± SD, [min, max])	6.86 ± 4.66, [2.72, 19.81]	8.57 ± 6.11, [2.43, 21.56]	0.322
Sex (*n*, %)			0.00421
Male	24, 75.0%	10, 37.0%	
Female	8, 25.0%	17, 63.0%	

**TABLE 2:** Stool metabolite concentrations (nM) measured by targeted LC-MS/MS in NAC and ASD participants.

Metabolite^a^	NAC (*N* = 27)			ASD (*N* = 32)		
	Mean (SD) [min, max]	Median	Below LOD, *n* (%)	Mean (SD) [min, max]	Median	Below LOD, *n* (%)
2-arachidonoylglycerol (2-AG)	13.35 (14.76) [4.64, 75.59]	8.08	0/27 (0%)	16.86 (18.9) [4.5, 98.97]	9.72	0/32 (0%)
3-nitrotyrosine (3-NT)	23.39 (13.91) [7.47, 67.25]	19.51	0/27 (0%)	25.55 (18.72) [11.09, 109.88]	20.2	0/32 (0%)
Acetylcholine (ACh)	291.87 (275.15) [39.07, 1,243.44]	198.25	0/27 (0%)	480.75 (647.52) [30.84, 3,601.75]	285.29	0/32 (0%)
Anandamide (AEA)	0.6 (0.5) [0.06, 2.04]	0.41	0/27 (0%)	0.91 (1.9) [0.07, 10.85]	0.48	0/32 (0%)
Butyric acid (BA)	175.94 (81.53) [64.59, 378.92]	152.16	0/27 (0%)	198.26 (72.28) [88.48, 344.98]	159.44	0/32 (0%)
Deoxycholic acid (DCA)	2,492.33 (2,814.79) [1.2, 14,089.73]	1,851.15	0/27 (0%)	2,970.46 (2,259.85) [10.72, 7,718.99]	2,407.29	0/32 (0%)
Dopamine (DA)	58.74 (5.34) [53.95, 75.09]	56.02	0/27 (0%)	59.26 (3.7) [54.39, 68.11]	58.87	0/32 (0%)
γ-aminobutyric acid (GABA)	1,336.64 (1,269.14) [147.65, 5,715.81]	1,156.47	0/27 (0%)	2,350.25 (6,380.55) [22.42, 36,728.33]	818.47	0/32 (0%)
Glycodeoxycholic acid (GDCA)	6.94 (18.51) [0.15, 94.76]	1.07	0/27 (0%)	13.74 (35.32) [0.22, 178.84]	2	0/32 (0%)
Indoleacetic acid (IAA)	1,435.3 (797.83) [232.69, 3,238.13]	1,236.12	0/27 (0%)	1,669.28 (1,601.87) [61.91, 7,981.73]	1,259.24	0/32 (0%)
Kynurenine (KYN)	216.84 (222.65) [35.49, 816.53]	114.45	0/27 (0%)	204.18 (274.8) [20.24, 1,541.99]	136.57	0/32 (0%)
Lithocholic acid (LCA)	2,460 (3,532.58) [21.09, 14,783.97]	990.51	1/27 (3.7%)	4,433.82 (6,503) [69.2, 25,907.6]	2,015.19	1/32 (3.12%)
L-tryptophan (Trp)	254.13 (202.57) [50.53, 992.11]	187.54	0/27 (0%)	1,183.54 (4,090.44) [36.68, 23,044.91]	240.38	0/32 (0%)
Nicotinic acid (NA)	274.64 (253.58) [14.51, 1,203.93]	220.25	0/27 (0%)	454.68 (359.91) [22, 1,375.45]	334.86	0/32 (0%)
Norepinephrine (NE)	5.1 (5.58) [0.84, 20.42]	2.42	8/27 (29.63%)	8.26 (12.86) [0.36, 63.91]	3.88	5/32 (15.62%)
Serotonin (5-HT)	41.4 (161.44) [1.34, 846.34]	4.1	0/27 (0%)	45.22 (117.53) [1.13, 603.58]	7.62	0/32 (0%)
Tetrahydrobiopterin (BH4)	80.61 (2.67) [76.39, 88.96]	80.05	0/27 (0%)	79.93 (2.21) [77.17, 84.67]	79.18	0/32 (0%)
Urolithin A (UA)	118.78 (444.34) [0.02, 2,180.57]	0.61	3/27 (11.11%)	40.68 (114.27) [0.01, 497.8]	0.31	2/32 (6.25%)

^a^Summary statistics (mean, SD, median, and range [min, max]) were computed after excluding measurements below the limit of detection (LOD). “Below LOD, *n* (%)” denotes the number and percentage of samples with concentrations below the LOD; these values were treated as zeros in downstream analyses.

### 3.1 Groupwise differences in stool metabolite abundance and statistical assessment

**FIGURE 2:**
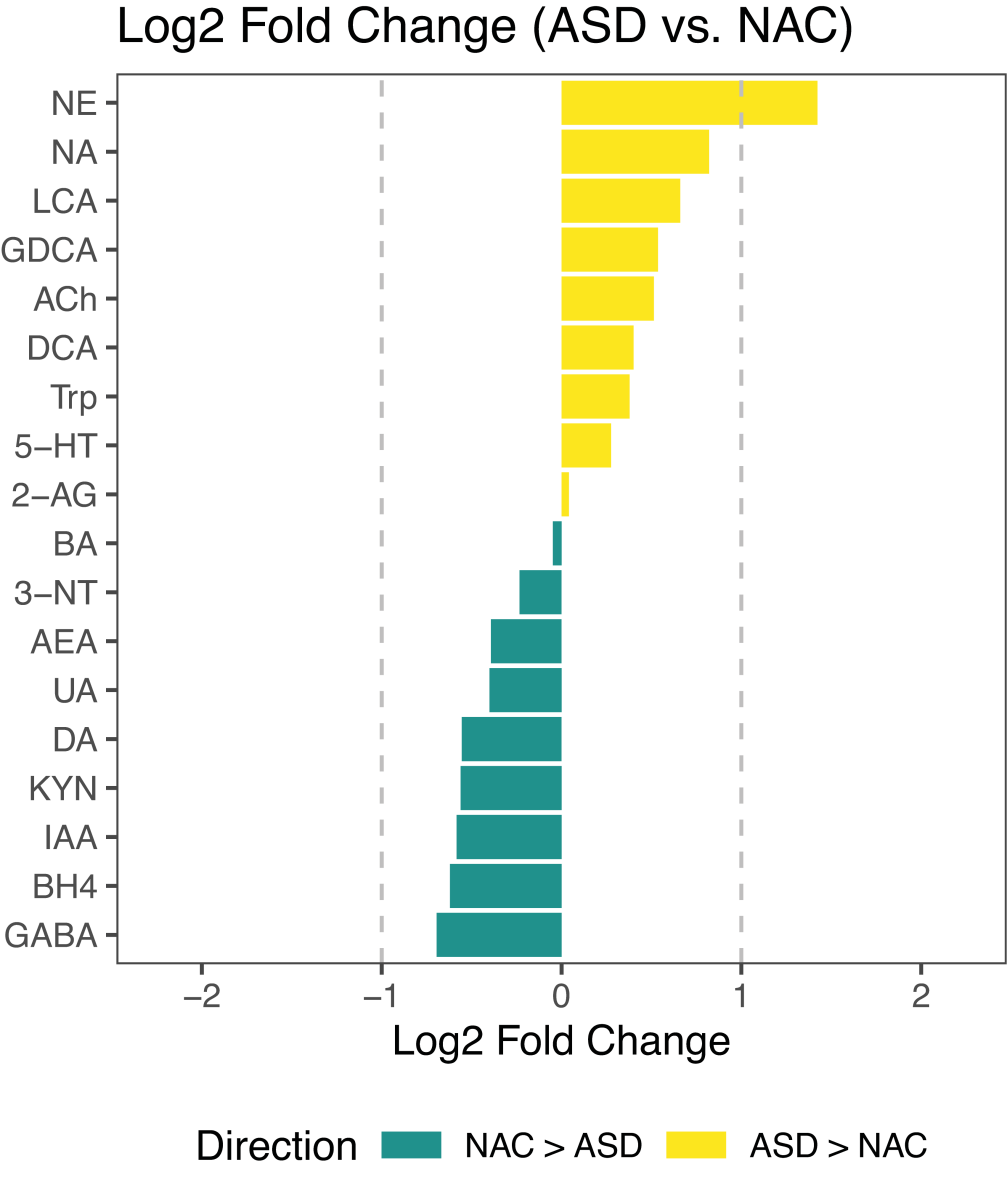
Log2 fold change of metabolite abundances between ASD and NAC groups. Positive values indicate higher abundance in the ASD group, while negative values indicate higher abundance in the NAC group. Dashed lines represent ±1 log2 fold change thresholds, corresponding to a two-fold difference in either direction.

Groupwise differences for each metabolite were assessed using log2 fold changes derived from replicate-averaged metabolite data after quality filtering, median normalization, log10 transformation, and Pareto scaling (Figure 2 and Supplementary Table 2). Norepinephrine exhibited the largest positive log2 fold change (1.42), indicating higher normalized signal in ASD, whereas BH4, DA, and GABA showed the largest negative log2 fold changes. We next evaluated statistical significance using linear models adjusted for age and sex. BH4 (uncorrected *p* = 0.020) and DA (uncorrected *p* = 0.031) showed nominal group differences, but no metabolite remained significant after false discovery rate correction (all q > 0.05). Thus, no individual metabolite showed statistically robust diagnostic group differences after multiple-testing correction, and these abundance-level findings should be interpreted as exploratory. Detailed model statistics are reported in Supplementary Table 2.

### 3.2 Discriminative performance of metabolite features in classifying ASD

**FIGURE 3:**
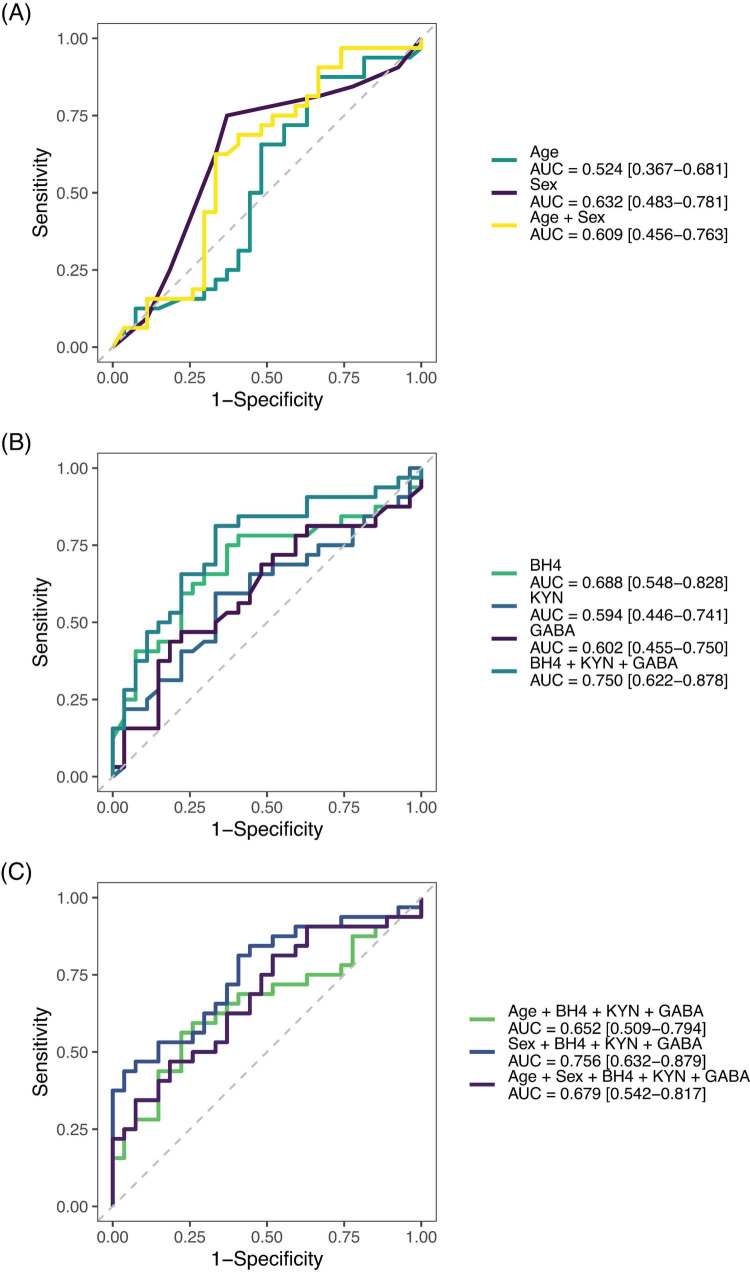
ROC curves for random forest models predicting ASD versus NAC across different predictor sets. Curves show five-fold cross-validated performance for models based on **(A)** demographic variables only, **(B)** individual and combined metabolite markers, and **(C)** combined demographic and metabolite predictors. AUC values and 95% confidence intervals are shown in the legend. ASD was defined as the positive class.

To evaluate the discriminative performance of fecal metabolites in classifying ASD, we first trained random forest models using each metabolite individually and evaluated them using five-fold cross-validation. The top three metabolites based on AUC were BH4 (AUC = 0.689 [0.549–0.829]), GABA (AUC = 0.606 [0.459–0.753]), and KYN (AUC = 0.595 [0.447–0.742]; Supplementary Table 3). We then constructed multivariate random forest models using these top-ranked features with and without demographic covariates (Figure 3 and Supplementary Table 4). Models based on age or sex alone showed limited discrimination. In contrast, the three-metabolite model combining BH4, KYN, and GABA achieved an AUC of 0.750 [0.622–0.878], with an accuracy of 0.746, an F1-score of 0.776, a precision of 0.743, a recall (sensitivity) of 0.812, and a specificity of 0.667. Adding sex to this model produced a modest increase in AUC to 0.757 [0.634–0.880], whereas adding age reduced AUC to 0.655 [0.514–0.796]. The full model incorporating age, sex, BH4, KYN, and GABA achieved an AUC of 0.679 [0.542–0.817]. To assess whether broader feature inclusion improved classification, we additionally compared models using the top three metabolites, the top three plus Trp, the top five metabolites, and all 18 measured metabolites (Supplementary Table 5). The three-metabolite model performed best, whereas inclusion of Trp or additional metabolites reduced AUC, supporting the use of a parsimonious feature set.

### 3.3 Model calibration performance

**FIGURE 4:**
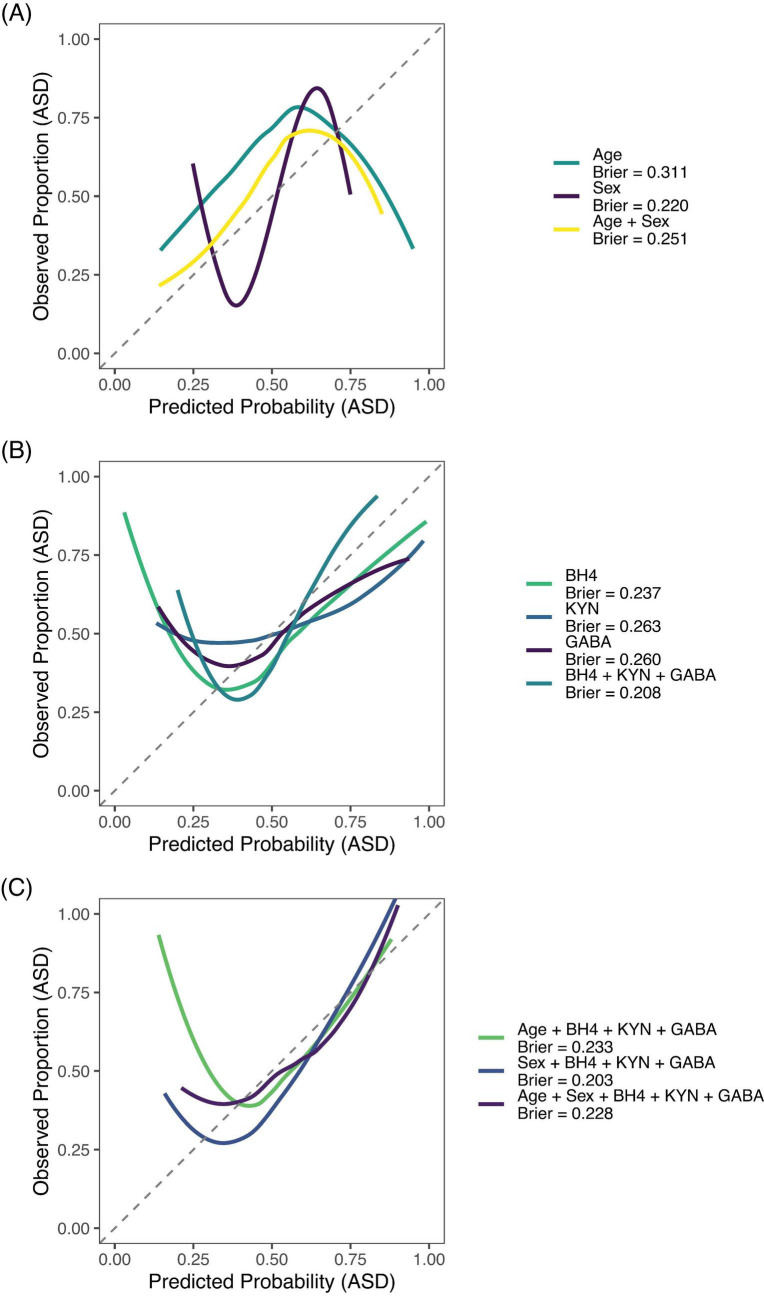
Calibration of random forest models across predictor sets. Calibration plots compare predicted probabilities of ASD classification with observed ASD proportions using LOESS-smoothed curves from five-fold cross-validation for models using **(A)** demographic variables only, **(B)** individual and combined metabolite markers, and **(C)** combined demographic and metabolite predictors. The gray dashed line indicates perfect calibration. Brier scores are shown in the legend.

To ensure that the predicted probabilities of the constructed random forest models reflected observed outcomes, we evaluated model calibration using the Brier score and LOESS-smoothed calibration curves (Figure 4). Lower Brier scores indicate better calibration. The model combining sex with the top three metabolites (BH4, KYN, and GABA) showed the best calibration, achieving the lowest Brier score of 0.203. This was followed by the three-metabolite model alone (Brier score = 0.208). The full model incorporating age, sex, and the three metabolites had a Brier score of 0.228, whereas the model combining age with the three metabolites had a Brier score of 0.233. Among demographic-only models, the sex-only model showed better calibration (Brier score = 0.220) than the age-only model (Brier score = 0.311), and the age + sex model had a Brier score of 0.251. Among individual metabolite models, BH4 showed better calibration (0.237) than KYN (0.263) and GABA (0.260). Full calibration scores for all models are reported in Supplementary Table 4.

### 3.4 Within-group correlation analysis of stool metabolites

**FIGURE 5:**
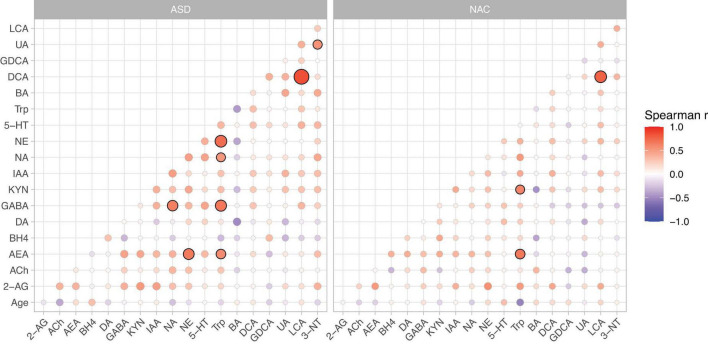
Within-group correlation matrices of stool metabolites and age in ASD and NAC. Spearman correlations are shown for ASD (left) and NAC (right). Circle color represents the correlation coefficient (r; red = positive, blue = negative), circle size indicates the strength of statistical evidence (–log_10_[q]), and significant correlations after FDR correction (q < 0.05) are highlighted with a black outline.

We next examined within-group correlations among stool metabolites and age using Spearman correlation. A total of eight significant correlations were identified in the ASD group and three in the NAC group, following FDR correction applied separately within the ASD and NAC groups (Supplementary Table 6). In both groups, LCA and deoxycholic acid (DCA) were strongly correlated (*r* = 0.85 in ASD; *r* = 0.78 in NAC). In the ASD group, additional significant correlations included Trp with GABA (*r* = 0.68), NA (*r* = 0.53), anandamide (AEA; *r* = 0.58), and NE (*r* = 0.72), as well as NE with AEA (*r* = 0.66), NA with GABA (*r* = 0.63), and 3-nitrotyrosine with UA (*r* = 0.55). In the NAC group, significant correlations were observed between Trp and AEA (*r* = 0.66) and between Trp and KYN (*r* = 0.61). The correlation matrices for each group are shown in Figure 5. To facilitate interpretation of the correlation network findings, all metabolite pairs that remained significant after FDR correction were visualized as scatterplots with corresponding Spearman correlation coefficients and FDR-adjusted q-values (Supplementary Figure 2).

## 4 Discussion

### 4.1 Summary of principal findings

We observed the largest positive log2 fold change for NE, whereas BH4 and DA showed nominal age- and sex-adjusted group differences that did not survive FDR correction. Random forest analysis identified BH4, GABA, and KYN as the most informative metabolites, and a parsimonious three-metabolite model outperformed larger feature sets. Correlation analyses revealed conserved LCA-DCA coupling and altered Trp-linked associations in ASD. A small subset of metabolites (LCA, NE, and UA) showed values at or below the LOD and manual inspection of MS signals confirmed this reflected true low abundance. Overall, the results suggest modest group-level metabolic differences and possible alterations in metabolic network structure in ASD.

These findings should be interpreted in the context of prior ASD metabolomics studies in blood, urine, and other biospecimens. Previous studies have reported alterations in tryptophan metabolism, kynurenine-pathway metabolites, catecholamine-related compounds, oxidative stress markers, and microbial co-metabolites, but results have varied substantially by cohort, analytical platform, and sample matrix (Gevi et al., 2020; Likhitweerawong et al., 2021; Needham et al., 2021; Almulla et al., 2023; Yildirim et al., 2023). This heterogeneity is important because fecal metabolites reflect a mixture of host, microbial, dietary, and luminal processes and may not directly mirror circulating or central nervous system metabolism (Zierer et al., 2018; Visconti et al., 2019; Needham et al., 2021).

### 4.2 Exploratory catecholamine-linked findings in ASD

The classical dopamine hypothesis of ASD attributes its core behavioral features to dysfunction within central dopaminergic circuits (Pavãl, 2017). Dopaminergic regulation also operates peripherally, and gut microbiome-mediated processes can influence catecholamine signaling via the GBA (Miri et al., 2023). In our dataset, BH4 and DA showed nominal reductions in ASD after adjustment for age and sex, whereas NE showed a larger positive log2 fold change. Because none of these differences survived FDR correction, these findings should be interpreted as exploratory signals rather than definitive evidence of catecholaminergic dysregulation.

The BH4 decrease is notable given its central role in monoamine synthesis and social behavior. As an essential cofactor for tyrosine hydroxylase and tryptophan hydroxylase, BH4 supports both catecholamine and serotonin biosynthesis, while also serving as a required cofactor for nitric oxide synthase activity (Werner et al., 2011; Fanet et al., 2021; Eichwald et al., 2023). Consequently, reduced BH4 availability may impair neurotransmitter production and synaptic signaling, positioning BH4 at the intersection of catecholamine and serotonin/melatonin-related biology (Werner et al., 2011; Pavãl, 2017). This connection is particularly relevant to ASD because elevated whole-blood serotonin remains one of the most consistently replicated peripheral biomarkers observed in a subset of affected individuals, and alterations in the serotonin/N-acetylserotonin/melatonin pathway have also been reported (Pagan et al., 2014; Muller et al., 2016; Esposito et al., 2024). However, because the present stool panel did not quantify serotonin, 5-hydroxytryptophan, N-acetylserotonin, melatonin, or platelet serotonin, the observed BH4 difference cannot be used to infer serotonin-pathway activity directly. Future studies should therefore evaluate serotonin- and melatonin-pathway intermediates alongside BH4, catecholamines, and Trp-KYN/NAD+-precursor metabolites to better characterize the broader metabolic consequences of altered BH4 availability. The biological relevance of BH4 in ASD is further supported by preclinical studies showing that L. reuteri restores social behavior in ASD mouse models by increasing BH4-pathway metabolites, whereas BH4 supplementation alone can reproduce this behavioral rescue (Buffington et al., 2021). Consistent with these findings, pediatric studies report improvements in social and behavioral domains with BH4 supplementation (Klaiman et al., 2013). Collectively, these observations are consistent with a potential role for BH4 as a mechanistic link between gut dysbiosis, altered monoamine metabolism, and social impairments in ASD.

The concurrent reductions in DA and BH4 align with BH4’s role as a cofactor for tyrosine hydroxylase (Fanet et al., 2021). By contrast, the elevation of stool NE diverges from this expected relationship, suggesting additional sources beyond host DA availability. One plausible explanation is partial microbial production independent of host catecholamine metabolism (Miri et al., 2023). Several gut bacteria can synthesize NE *de novo* through enzymes analogous to dopamine β-monooxygenase (Miri et al., 2023). NE also modulates microbial quorum sensing, reinforcing bidirectional host-microbe signaling (Miri et al., 2023). Interpretation of fecal catecholamines is also complicated by host phase II metabolism, including sulfoconjugation, deamination, and O-methylation, which influence catecholamine bioavailability, clearance, and measured abundance (Rivett et al., 1982; Goldstein, 2010). These conjugated catecholamine species and related enzymatic processes were not measured in the present targeted panel, limiting inference about catecholamine turnover.

Taken together, these findings are consistent with the possibility that BH4-dependent catecholamine biology and microbial catecholamine activity contribute to the observed stool metabolite pattern. However, because the group differences did not survive FDR correction and microbial composition was not measured, these interpretations remain speculative. Future studies could evaluate whether interventions targeting BH4 availability or microbial catecholamine synthesis influence gut catecholaminergic profiles or ASD-relevant phenotypes (Clarke et al., 2006; Frye et al., 2013; Liu et al., 2022).

### 4.3 Altered tryptophan-linked and NAD+-precursor associations

To explore alterations in metabolic pathways associated with gut microbial activity, we examined within-group correlations among fecal metabolites. A robust positive correlation between LCA and DCA was observed in both groups, consistent with their shared microbial origin (Kiriyama and Nochi, 2021). Beyond this conserved bile acid relationship, prominent group-specific differences involved Trp-linked metabolic associations. In controls, Trp correlated strongly with KYN, consistent with coordinated activity of the hepatic KYN pathway (Badawy, 2017; Dehhaghi et al., 2019). However, this coupling was absent in the ASD group, suggesting a possible difference in Trp-KYN coupling. Instead, Trp formed additional associations with NA, GABA, NE, and AEA.

The Trp-NA association is noteworthy because both metabolites are integral to NAD+ biosynthesis. NAD+ can be produced *de novo* from Trp through the KYN pathway or from NA via the Preiss-Handler deamidated route (Xie et al., 2020). Microbial nicotinamidase (PncA) can bypass host constraints by converting nicotinamide (NAM) to NA, feeding into the Preiss-Handler pathway (Shats et al., 2020). Since mammalian cells lack the ability to deamidate NAM, this microbial reaction creates a unique host-microbe interface in NAD+ metabolism.

Microbial participation in NAD+ precursor metabolism provides one possible interpretation of the Trp-NA association observed in ASD. One possibility is that this correlation reflects altered microbial participation in NAD+ precursor cycling, potentially as a compensatory response to maintain NAD+ levels under conditions of disrupted Trp-KYN metabolism or oxidative imbalance. Alternatively, shifts in the abundance or activity of PncA-expressing taxa could drive excessive deamidation, favoring NA-dependent NAD+ biosynthesis over canonical host routes. Collectively, these observations are consistent with a possible host-microbe metabolic relationship involving NAD+ precursor metabolism and gut microbial activity in ASD. Importantly, because the present study did not directly measure NAD+, NADH, nicotinamide, microbial PncA activity, or pathway flux, any mechanistic inference remains tentative.

### 4.4 Discriminative value of stool metabolites

Motivated by the catecholamine- and Trp-linked patterns described above and the feasibility of stool as a pediatric, noninvasive sample, we assessed whether stool metabolites carry discriminative signal for ASD. Prior work suggests that gut-microbiome features can aid ASD screening and subtype stratification (Kong et al., 2019, 2021; Su et al., 2024; Wan et al., 2024). Building on this foundation, we extend from taxa-centric to metabolite-centric profiling, offering a complementary biological readout that may help contextualize behaviorally defined ASD phenotypes. Among the profiled metabolites, BH4, GABA, and KYN were the most informative and map to non-redundant biology, which likely explains their combined gain in discrimination. BH4 captures cofactor availability for monoamine synthesis and aligns with the catecholaminergic findings above (Werner et al., 2011); GABA reflects inhibitory neurotransmission that can be produced and modulated by gut microbes (Braga et al., 2024); and KYN marks immune-regulated Trp catabolism, consistent with the altered Trp-linked correlations we observed (Badawy, 2017; Dehhaghi et al., 2019). Although biologically informative, the observed AUC reflects only modest discrimination in a small, internally cross-validated cohort. Therefore, this panel should not be interpreted as having diagnostic utility without replication in larger, independent samples.

Each single-metabolite model showed only modest discrimination (AUC < 0.7), indicating that no single compound is sufficient. In contrast, the three-metabolite model showed moderate discrimination with high recall for ASD and acceptable calibration. Notably, expanding the feature set to include Trp, the top five metabolites, or all 18 measured metabolites did not improve performance, suggesting that the primary discriminatory signal was captured by a small subset of non-redundant features rather than by diffuse information across the full panel.

The additional boost when sex was included is directionally consistent with known sex differences in ASD prevalence and gut-brain physiology, (Kopec et al., 2018) although it may also partly reflect cohort imbalance. By contrast, age reduced discrimination, likely due to the cohort’s uneven age structure, which introduces variance without stable diagnostic signal. Overall, the multi metabolite panel should be viewed as a mechanistically coherent, hypothesis-generating signature rather than a validated diagnostic tool, particularly because hyperparameter tuning and external validation were not performed.

### 4.5 Study limitations and future directions

Several limitations of this study should be acknowledged. First, the sample size was modest, which limits statistical power to detect small-to-moderate effects. The modest sample size is particularly relevant for the machine-learning analyses, where model instability and optimistic performance estimates may occur despite the restricted targeted metabolite panel and use of stratified cross-validation. Therefore, the random forest results should be interpreted as exploratory evidence of potential discriminative signal rather than as a validated classification model. While multiple metabolites showed differences between groups, none survived false discovery rate correction, and the findings should therefore be considered exploratory. Second, the cross-sectional design precludes causal inference or assessment of developmental trajectories, which require longitudinal follow-up. Accordingly, the present data cannot determine whether the observed metabolite patterns contribute to ASD-related biology, arise as downstream consequences of ASD-associated behavioral, dietary, gastrointestinal, or microbial differences, or reflect unrelated cohort-specific variation. Third, several important determinants of the stool metabolome, including diet, gastrointestinal symptoms, medication exposure, and microbiome composition, were not directly measured or incorporated into the statistical models. Although recent antibiotic, probiotic, and oxytocin use were exclusionary and medication regimens were required to be stable, unmeasured medication class, dietary intake, gastrointestinal comorbidity, and microbial taxonomic variation may have influenced metabolite concentrations (Zierer et al., 2018; Needham et al., 2021; Shinn et al., 2022). Pre-analytical and analytical factors (collection timing, storage, matrix effects, batch variation) may also introduce variability. Although below-LOD values were confirmed by manual inspection to reflect true low-abundance signals, retaining them as zeros in downstream analyses may influence distributional assumptions, effect-size estimates, and machine-learning performance. Alternative approaches for handling below-LOD observations, including half-minimum substitution, left-censored imputation methods, and other missing-data strategies, should be evaluated in future validation cohorts (Do et al., 2018; Wei et al., 2018). The cohort’s age structure and sex imbalance may confound associations. Although sex was included as a covariate in the linear models and was evaluated in the machine-learning analyses, residual confounding remains possible because statistical adjustment cannot fully compensate for imbalance in a modestly sized cohort. The machine-learning models also require external validation. In addition, participants with ASD were recruited via clinician referral and specialty clinical programs, whereas non-ASD controls were recruited from community sources. This convenience sampling strategy may introduce selection bias and limit the representativeness of both groups relative to the broader pediatric population. Differences in healthcare-seeking behavior, socioeconomic factors, or unmeasured environmental exposures between recruitment sources may further influence observed metabolic patterns despite statistical adjustment for age and sex.

Future studies should incorporate larger, independent cohorts and longitudinal sampling to assess developmental trajectories. Integrative multi-omics approaches may clarify mechanisms linking metabolic alterations to ASD, and translational work is needed to evaluate stool metabolites as candidate adjunctive biomarker features and to test targeted metabolic or microbiome-based interventions (Frye et al., 2013; Klaiman et al., 2013; Filho et al., 2025).

Despite these limitations, this exploratory study provides a focused assessment of stool metabolites linked to catecholamine biology, inhibitory neurotransmission, and tryptophan-related metabolism in ASD. Although no individual metabolite survived FDR correction, the findings suggest potentially altered metabolite associations involving Trp, pterin, catecholamine, and NAD+-precursor biology. These results should be considered hypothesis-generating and require replication with larger cohorts, deeper phenotyping, microbiome profiling, and external validation of candidate classification models.

## Data Availability

The raw data supporting the conclusions of this article will be made available by the authors, without undue reservation.
