# Supplementary material for: Targeted stool metabolomics suggests exploratory catecholamine- and tryptophan-linked metabolic features in autism spectrum disorder
Source: Front Neurosci. 2026 Jul 3;20:1858005. doi: 10.3389/fnins.2026.1858005 (PMC13375968; doi:10.3389/fnins.2026.1858005)
Supplement: Supplementary file 7 [file Data_Sheet_1.docx]

**Supplementary Figure 1.** Age Distribution of Study Participants by Diagnostic Group.
